# Simultaneously Dealing With Immortal Time Bias and Residual Confounding: A Case Study of a High‐Dimensional Propensity Score Approach With a Nested Case–Control Framework in Multiple Sclerosis Research

**DOI:** 10.1002/pds.70174

**Published:** 2025-06-24

**Authors:** Md. Belal Hossain, Huah Shin Ng, Feng Zhu, Helen Tremlett, Mohammad Ehsanul Karim

**Affiliations:** ^1^ School of Population and Public Health University of British Columbia Vancouver British Columbia Canada; ^2^ Centre for Advancing Health Outcomes University of British Columbia Vancouver British Columbia Canada; ^3^ College of Medicine and Public Health, Flinders University Bedford Park South Australia Australia; ^4^ SA Pharmacy SA Health Adelaide South Australia Australia; ^5^ Division of Neurology, Department of Medicine The Djavad Mowafaghian Centre for Brain Health, University of British Columbia Vancouver British Columbia Canada

**Keywords:** disease‐modifying drugs, high‐dimensional propensity score, immortal time bias, multiple sclerosis, nested case–control, residual confounding, time‐dependent exposure

## Abstract

**Background:**

Observational studies of time‐dependent treatments often face immortal time bias and residual confounding, complicating treatment effect estimation. We implemented a high‐dimensional propensity score (hdPS) analysis within a nested case–control (NCC) framework to address both biases simultaneously.

**Methods:**

We used a retrospective cohort of 19 360 individuals with multiple sclerosis (MS) in British Columbia, Canada, to examine the relationship between disease‐modifying drugs (DMDs) and all‐cause mortality. A 1:4 NCC analysis addressed immortal time bias, and hdPS was applied to handle residual confounding. Sensitivity analyses tested the robustness of findings across various hdPS parameters and matching strategies.

**Results:**

We matched a total of 3209 cases to 12 293 controls in the NCC analysis, and demonstrated a 28% reduction in mortality risk associated with exposure to DMDs (hazard ratio [HR]: 0.72, 95% confidence interval [CI]: 0.62–0.84) in the NCC‐hdPS analysis. Sensitivity analyses using different propensity score estimation techniques and control‐matching strategies yielded consistent results, with HRs ranging between 0.70 and 0.77.

**Conclusions:**

This study offers a practical framework for addressing immortal time bias and residual confounding simultaneously, improving the validity of effect estimates in real‐world studies. We shared reproducible R codes for researchers to facilitate the adoption of this methodology in their research.

**Summary**

- The manuscript presents a framework for employing a high‐dimensional propensity score method within a nested case–control design to simultaneously address immortal time bias and residual confounding in observational studies.
- Applied to a cohort of individuals with multiple sclerosis, the study demonstrates that disease‐modifying drugs reduce all‐cause mortality by 28%.
- The inclusion of reproducible software code will assist future researchers in applying this framework to enhance the validity of treatment effect estimates in real‐world settings.

## 1 Introduction

### 1.1 Immortal Time Bias in Observational Studies

Observational studies using large health administrative databases provide valuable insights into the real‐world effects of a treatment. However, these studies are prone to several methodological challenges, notably immortal time bias [1, 2]. Immortal time bias occurs when a period during follow‐up, in which a patient cannot experience the outcome (e.g., death), is misclassified as time under observation, often leading to an overestimation of the treatment's effectiveness [2, 3]. As an example, in MS research exploring the relationship between disease‐modifying drugs (DMDs) and long‐term mortality, immortal time can occur if patients must survive long enough to receive a DMD, which could falsely enhance the perceived survival benefit of these drugs. Previous studies have mitigated this bias by employing Cox proportional hazards models with time‐varying exposure to DMDs [4, 5, 6].

### 1.2 Residual Confounding in Observational Studies

While the time‐varying Cox proportional hazards model helps mitigate immortal time bias, it does not eliminate the risk entirely, especially if confounding factors are not fully accounted for, leading to residual confounding. In the context of MS research, key factors such as lifestyle behaviors and MS severity are often inconsistently captured in administrative data, which could bias estimates of the true effect of DMDs on survival. A recent study highlighted that, despite using robust statistical models, their findings were still subject to potential residual confounding due to limitations in capturing certain variables accurately [7]. This highlights the need for more advanced methodological approaches to account for residual confounding. One such approach is the high‐dimensional propensity score (hdPS) method, which systematically incorporates a large number of proxy variables from health administrative data, helping to minimize bias due to residual confounding.

### 1.3 Challenges in Simultaneously Dealing With Immortal Time Bias and Residual Confounding

The hdPS approach is used to deal with residual confounding with a binary “time‐fixed” treatment [8, 9, 10]. However, since treatment is a “time‐dependent” variable in the time‐varying Cox proportional hazards model, implementing the hdPS framework in conjunction with a time‐varying Cox proportional hazards model presents a methodological and practical challenge. To address this limitation, the current study leverages a nested case–control (NCC) design. The NCC framework provides a robust alternative for addressing immortal time bias while allowing for the integration of hdPS analysis to minimize the residual confounding [11, 12].

### 1.4 Additional Challenge With a Time‐to‐Event Outcome

The hdPS technique deals with residual confounding in seven well‐defined steps [8, 9]. The Bross formula is commonly used to prioritize the proxy (recurrence) covariates created from the proxy variables. However, the Bross formula requires the exposure, outcome, and proxy covariates to be binary [8]. Ignoring the time aspect in a time‐to‐event outcome leads to a loss of information, which can impact both the selection of proxy covariates and the effect estimate of interest. Regularized regression such as Least Absolute Shrinkage and Selection Operator (LASSO) for time‐to‐event outcomes could be useful in prioritizing and selecting recurrence covariates. A previous study with a binary outcome showed that LASSO prioritization and selection of recurrence covariates could better address residual confounding than the Bross‐based approach [13].

### 1.5 Study Aim

Our aim was to conduct a case study examining the relationship between DMDs for MS and all‐cause mortality, while addressing both immortal time bias and residual confounding. This case study allowed us to detail the implementation of the hdPS approach within an NCC framework. A novel aspect of our work was the application of a LASSO model for time‐to‐event outcomes to prioritize and select proxy covariates. We also performed a series of sensitivity analyses to fine‐tune key hdPS parameters and assess the robustness of our findings.

## 2 Methods

### 2.1 Case Study Description

#### 2.1.1 Study Setting and Population

We utilized data from a retrospective cohort of individuals with MS in British Columbia, Canada, between January 1, 1996, and December 31, 2017 (Appendix A). The cohort was developed based on public health surveillance and linked health administrative databases [4]. Data elements include demographics (demographic information including three‐digit postal codes to derive neighborhood‐level income using Statistics Canada's algorithm) [14], vital statistics (deaths in British Columbia) [15], medical services plan (registration and physician billings) [16, 17], hospital discharge abstract database (inpatients and day surgeries) [18], and Pharmanet (prescription dispensations in outpatient or community pharmacies) [19]. These data sources were integrated using unique patient identifiers, ensuring comprehensive and accurate linkage across demographic, clinical, and pharmaceutical records. The authors did not have access to any information that could directly identify individual participants during or after data collection. Ethical approval of the study was provided by the University of British Columbia (H1800407).

#### 2.1.2 Index Date

The index date (representing the start of follow‐up) was the most recent clinically relevant event, such as first clinical evidence of MS or related demyelination (ICD‐9: 340, ICD‐10: G35), MS DMD initiation, the person's 18th birthday, or January 1, 1996 [4, 20]. A 1‐year residency in the province (defined as ≥ 90% of the days) before the index date for each individual enabled characterization of the comorbidity burden at the index date [20].

#### 2.1.3 Outcome

All‐cause mortality was chosen as the primary outcome to determine the association between DMD exposure and long‐term survival in MS patients [4, 20]. The follow‐up time, measured in years, was calculated from the index date to the earliest of death, emigration from the province (measured as the date of cancelation of the mandatory provincial health insurance plan), or December 31, 2017 (end of the study).

#### 2.1.4 Exposure

We defined the exposure as cumulative use of any DMD based on prescription data. Specifically as: ≥ 180 days of cumulative use for beta‐interferon or glatiramer acetate, or ≥ 90 days of cumulative use for natalizumab, fingolimod, dimethyl fumarate, or teriflunomide, guided by prior studies [21, 22, 23, 24]. The duration of DMD exposure was measured using the days supplied. Gaps in DMD supply for the same DMD class of ≤ 30 days were allowed when calculating contiguous exposure [4].

#### 2.1.5 Investigator‐Specified Covariates

Covariates were identified using a one‐year covariate assessment window before the cohort entry date. We considered the following covariates [4, 20, 25, 26] at the index date: age (continuous), sex (male/female), neighborhood income quintiles, comorbidity status (0, 1, 2, ≥ 3 comorbidities present), and calendar year (1996–1999, 2000–2005, 2006–2011, or 2012–2017). Comorbidity burden was assessed using a modified weighted Charlson Comorbidity Index (CCI) constructed using the physician claims and hospital discharge abstract databases [27]. Hemiplegia/paraplegia was excluded from the CCI to avoid misclassifying MS‐related neurological deficits as a comorbidity, which could skew the comorbidity burden of MS patients [25, 26].

#### 2.1.6 Statistical Analyses

##### 2.1.6.1 Nested Case–Control Design

To deal with immortal time bias, we designed an NCC study that matched subjects who experienced the event of interest (called cases) to a subset of event‐free subjects (called controls) using incidence density sampling [28]. As per the design, some controls could later become cases themselves and also serve as controls for other cases. In our NCC analysis, we matched each case (all‐cause mortality event) to up to four controls who were still alive (“1:4 NCC analysis”). We additionally matched the cases to controls by neighborhood income quintile, CCI, and calendar year [29]. Four controls per case were selected, as this has been shown to provide near‐optimal statistical efficiency without the need for the full cohort analysis [12]. We determined whether cases and matched controls were DMD exposed before the case's event time, ensuring comparable follow‐up duration between cases and controls [11, 12]. The conditional logistic regression was fitted with the matching criteria as the strata variable, adjusting for age and sex [12]. The hazard ratio (HR) with a 95% confidence interval (95% CI) was reported [30].

##### 2.1.6.2 hdPS in the NCC Framework

The time‐dependent exposure status becomes a “time‐independent” exposure variable in the NCC analysis [11, 12]. Hence, we could implement the hdPS technique in the NCC framework to allow for the systematic incorporation of a large number of empirical covariates, improving residual confounding adjustment by capturing a wider array of variables that might influence treatment assignment [8]. Thus, we implemented an NCC design to deal with immortal time bias, while the hdPS technique was integrated to deal with residual confounding, after adjusting for investigator‐specified covariates.

The steps for the hdPS analysis are as follows: (i) identify the data sources for the proxy covariates, (ii) identify the most prevalent proxy covariates, (iii) assess the recurrence of proxy covariates, (iv) prioritize the recurrence covariates, (v) select recurrence covariates, (vi) estimate propensity scores, and (vii) fit the outcome model [8, 9]. First, we used an additional three data sources to obtain proxy covariates with a 1‐year covariate assessment period prior to the index date: (i) physician visits: three‐digit International Classification of Diseases (ICD)‐9 diagnostic codes, (ii) hospitalizations: 3‐digit ICD‐9/10 diagnosis codes and procedure codes, and (iii) prescriptions dispensed: drug identification numbers. To avoid double counting, we omitted ICD‐9/10 codes that were used to define the CCI or MS as well as the drug identification numbers for the MS DMDs. Second, we considered the 1000 most prevalent proxy covariates in each of the four data dimensions. Third, each proxy covariate was converted to three binary empirical covariates based on their recurrence: (i) covariate was recorded ≥ once, (ii) covariate was recorded ≥ the median, and (iii) covariate was recorded ≥ the 75th percentile. Fourth, LASSO regularization with the Cox proportional hazards models (Cox‐LASSO) was employed for modeling the time‐to‐death survival outcome to prioritize and select covariates by shrinking less important recurrence covariates, which helps in reducing overfitting and improving model prediction [13]. We selected the lambda hyperparameter of the Cox‐LASSO model using fivefold cross‐validation and chose the hyperparameter that resulted in the minimum prediction error. Fifth, the Top 200 recurrence covariates were selected based on the absolute log‐HR of the covariates. Sixth, to estimate the propensity scores, where the exposure is a time‐fixed binary variable, we fitted a logistic regression model with LASSO regularization, with the following covariates: investigator‐specified confounders (age, sex) and the Top 200 recurrence covariates. Seventh, the inverse probability of treatment weighting (IPTW) approach was used to explore the relationship between DMD exposure and mortality [31]. Truncating extreme weights at the 99th percentile helps prevent model instability and reduces the potential influence of extreme weights, which can bias the treatment effect estimates [32]. The balance in the investigator‐specified covariates in the pseudo population was checked using standardized mean difference (SMD). We considered the SMD ≤ 0.2 as a good covariate balance [33]. Any investigator‐specified covariates showing SMD > 0.2 were adjusted in the outcome model (conditional logistic regression with the matching criteria as the strata variable). White's robust sandwich estimator was used to calculate the standard error (SE) [34]. To demonstrate how to apply the above‐described steps in a given scenario, reproducible R codes on a simulated dataset are provided in GitHub folder: https://github.com/belalanik/hdPS_NCC.

##### 2.1.6.3 Sensitivity Analyses

We conducted a series of sensitivity analyses for the pivotal decisions related to hdPS parameters, including (a) selecting an appropriate cut‐point for the prevalence filter to ensure stability in the treatment effect estimates, (b) investigating the robustness of results to different numbers of recurrence covariates in the propensity score model, (c) investigating the robustness of results across different data dimensions or sources included in the analysis, (d) deciding on the granularity of the ICD codes, (e) adding essential interaction and/or polynomial terms to both the hdPS and outcome models, and (f) determining if the propensity score model needs adjustment based on the covariate balance in the weighted data [8, 10, 13, 35]. By varying the prevalence cut‐points and the number of covariates, we assessed the robustness of the treatment effect estimates, ensuring that the final model was not overly sensitive to these selection thresholds. For (a), we considered the Top 200 codes from each data source or dimension based on prevalence compared to more than 1000 codes from each data source in the primary analysis. For (b), compared to the Top 200 recurrence covariates selected based on the absolute log‐HR from the Cox‐LASSO in the primary analysis, we considered (b.i) the top 100 and (b.ii) the top 500 recurrence covariates based on the absolute log‐HR of the covariates. Random survival forest was explored as an alternative to Cox‐LASSO for recurrence covariate selection, as it allows for the non‐parametric assessment of variable importance in survival outcomes. Therefore, we also considered (b.iii) the Top 100 and (b.iv) the Top 500 recurrence covariates identified using random survival forest [36], where we fitted the random survival forest model, calculated the variable importance measure using the Gini impurity index [37], and ranked the covariates based on the variable importance measure. For (c), we observed that the proportion of nonzero log‐HRs was highest for the physician visit‐related ICD codes (54.6%) in comparison to prescription dispensation (31.9%), hospital‐related ICD codes (12.4%), and hospital procedure‐related ICD codes (1.2%). We conducted the hdPS analysis using only the top 200 recurrence covariates from the physician visit data source. For (d), we considered four‐digit codes as opposed to the three‐digit codes used in the primary analysis. For (e), we used XGBoost to fit the hdPS model, as it flexibly captures the complex relationships (e.g., interactions or non‐linearities) among covariates without the need to prespecify terms such as age–sex interaction; avoiding reliance on potentially arbitrary interaction choices and allowing for a more data‐driven approach (see Appendix B) [38]. For (f), we considered SMD ≤ 0.1 as a good covariate balance compared to 0.2 used in the primary analysis [33, 39].

We conducted two sensitivity analyses to address the selection of controls per case. First, we considered up to eight controls per case (“1:8 NCC analysis”) to potentially improve the precision of the HR estimate [12]. Second, the NCC analysis could produce a noisier estimate due to the matching mechanism of the control selection. To reduce the variability and improve the stability of the HR calculation, we repeated the NCC analysis 100 times and averaged the results, providing more stable pooled estimates [40]. The modified multiple outputation rule, which combines within and between variance estimates, was used to provide a more stable and reliable calculation of model‐based SEs in the repeated NCC analyses [40]. A further sensitivity analysis was done with a full‐cohort time‐varying Cox proportional hazards model that helps mitigate immortal time bias but not residual confounding (Appendix B).

## 3 Results

### 3.1 Cohort Characteristics

There were 19 360 individuals with MS, with 225 930 person‐years of follow‐up (Table 1). The mean (SD) age was 44.5 (13.5) years; 72.0% were female, and 22.3% had at least one comorbidity. A total of 4211 (21.8%) individuals were exposed to any DMD, and 3210 individuals died during the follow‐up. Individuals who died during the study period, compared to those censored, were on average older (mean age 57.3 vs. 42.0 years) and more likely to have multiple comorbidities (31.0% vs. 20.5%).

**TABLE 1:** Characteristics of the study participants in exploring the relationship between exposure to any disease‐modifying drug (DMD) for multiple sclerosis and all‐cause mortality in British Columbia, Canada, 1996–2017, stratified by mortality status.

Characteristics	Total (*N* = 19 360)	Died (*N* = 3210)	Did not die during follow‐up and were censored (*N* = 16 150)
Follow‐up in years	225 930	31 496	194 434
Any DMD, *n* (%)	4211 (21.8)	228 (7.1)	3983 (24.7)
Age in years, mean (SD)	44.52 (13.54)	57.28 (14.33)	41.98 (11.84)
Sex, *n* (%)			
Female	13 940 (72.0)	1969 (61.3)	11 971 (74.1)
Male	5420 (28.0)	1241 (38.7)	4179 (25.9)
Neighborhood income quintile, *n* (%)			
Lowest 20%	3763 (19.4)	729 (22.7)	3034 (18.8)
Lower 20%	3695 (19.1)	655 (20.4)	3040 (18.8)
Middle 20%	4029 (20.8)	639 (19.9)	3390 (21.0)
Higher 20%	4094 (21.1)	635 (19.8)	3459 (21.4)
Highest 20%	3779 (19.5)	552 (17.2)	3227 (20.0)
Charlson comorbidity index, *n* (%)			
0	15 051 (77.7)	2216 (69.0)	12 835 (79.5)
1	2979 (15.4)	576 (17.9)	2403 (14.9)
2	855 (4.4)	244 (7.6)	611 (3.8)
≥ 3	475 (2.5)	174 (5.4)	301 (1.9)
Calendar index, *n* (%)			
1996–1999	8533 (44.1)	2377 (74.0)	6156 (38.1)
2000–2005	3905 (20.2)	477 (14.9)	3428 (21.2)
2006–2011	3722 (19.2)	272 (8.5)	3450 (21.4)
2012–2017	3200 (16.5)	84 (2.6)	3116 (19.3)

Abbreviations: DMD, disease‐modifying drugs; SD, standarad deviation.

### 3.2 Results From the NCC Analysis Without hdPS

**FIGURE 1:**
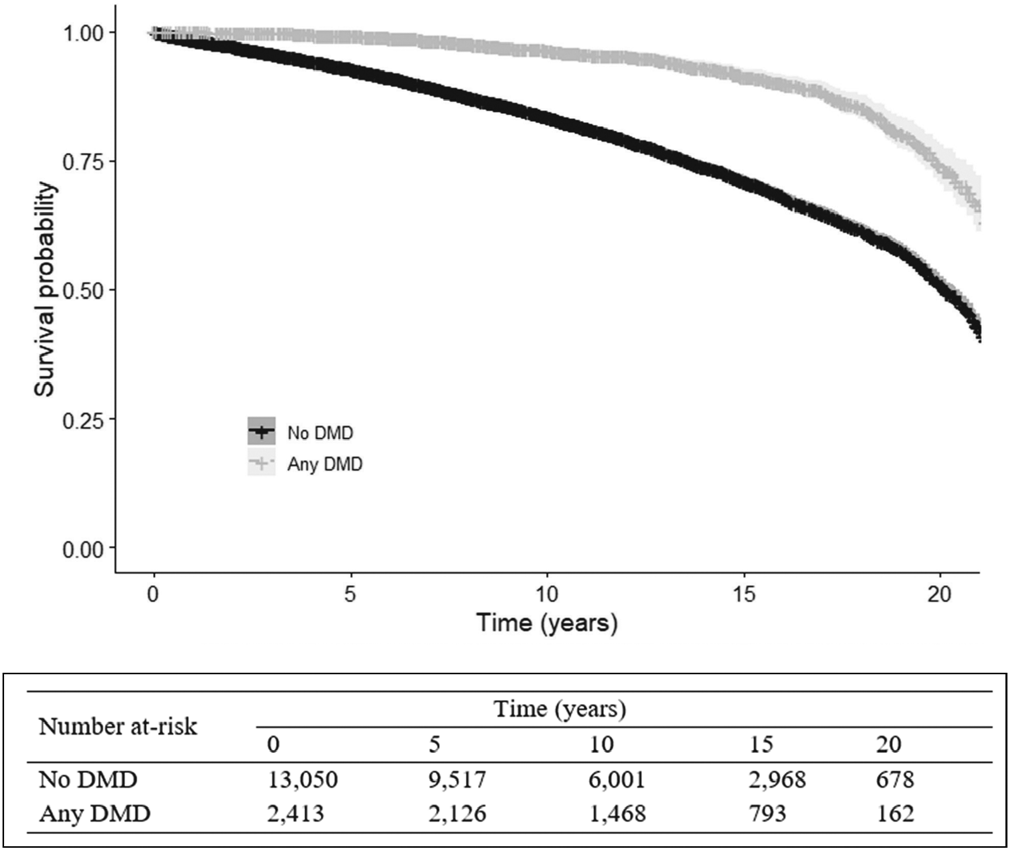
Kaplan–Meier curve in exploring the relationship between exposure to any disease‐modifying drug (DMD) for multiple sclerosis and all‐cause mortality in British Columbia, Canada, 1996–2017.

In the NCC analysis with four controls per case, 3209 cases were matched to 12 293 controls, while there was no suitable control for one case. The proportion of individuals by neighborhood income quintile, CCI, and calendar year was expectedly similar among cases and controls, since we matched the cases to controls by these covariates (Appendix Table 1). The Kaplan–Meier curve in Figure 1 showed a significantly higher survival probability in those exposed versus unexposed to any DMD (log‐rank test *p*‐value < 0.001). In the covariate‐adjusted analysis, where we adjusted the outcome model for age and sex and stratified by the matched set, the mortality risk was 28% less in persons with MS exposed to any DMD (HR: 0.72, 95% CI: 0.61–0.83, SE: 0.078) (Table 2).

**TABLE 2:** Relationship between exposure to any disease‐modifying drug (DMD) for multiple sclerosis and all‐cause mortality in British Columbia, Canada, 1996–2017.

Analysis	HR	SE	95% CI
Nested case control with four controls per case			
Covariate‐adjusted, non‐hdPS analysis^a^	0.72	0.078	0.61–0.83
hdPS with inverse probability weight^b^	0.72	0.077	0.62–0.84

Abbreviations: CI, confidence interval; hdPS, high‐dimensional propensity score; HR, hazard ratio; SE, standard error.

^a^ The conditional logistic regression on the matched dataset (matched using incidence density sampling) was fitted, with matched set as the strata variable, adjusting for age and sex.

^b^ The conditional logistic regression on the matched dataset (matched using incidence density sampling) was fitted, with the stabilized inverse probability weight, matched set as the strata variable, adjusting for age. The high‐dimensional propensity score technique was used to estimate the inverse probability weight. The mean of the untruncated stabilized weights was 1.47, while the mean of the truncated weights was 1.07 (minimum: 0.20, maximum: 2.04). Weights were truncated at the 99th percentile.

### 3.3 Results From the NCC Analysis With hdPS

In the NCC analysis with four controls per case where we implemented the hdPS technique, the truncated stabilized weights had a mean value close to 1, indicating appropriate weight truncation to avoid extreme values. However, age was imbalanced in terms of SMD in the inverse probability‐weighted pseudo‐population. Therefore, we adjusted the outcome model for age and matched strata, resulting in an HR of 0.72, with a 95% CI of 0.62–0.84 (Table 2).

### 3.4 Results From Sensitivity Analyses

#### 3.4.1 Sensitivity Analyses for hdPS Parameters

The results of sensitivity analyses for different hdPS parameters were not materially different compared to the non‐hdPS or primary hdPS analysis where four controls per case were selected (Table 3A). The hdPS analyses where the top recurrence covariates were identified using random survival forest also had a similar effect estimate. The mean of truncated stabilized weight was approximately 1 in all analyses. XGBoost was used as an alternative method for estimating propensity scores due to its ability to model complex relationships. This resulted in a slightly lower HR of 0.70 (95% CI: 0.60–0.82), with comparable SEs. All other sensitivity analyses yielded an HR of 0.72–0.73 with comparable SEs (0.076–0.078).

**TABLE 3:** Sensitivity analyses in exploring the relationship between exposure to any disease‐modifying drug (DMD) for multiple sclerosis and all‐cause mortality in British Columbia, Canada, 1996–2017.

Analysis	HR	SE	95% CI	Weight summary^a^					
				Min	Q1	Q2	Mean	Q3	Max
(A) Nested case control with four controls per case									
1. Analyses for hdPS parameters^b^									
(a) 200 proxy covariates from each data source	0.72	0.077	0.62–0.83	0.22	0.89	0.97	1.07	1.12	2.07
(b.i) Top 100 empirical covariates using Cox‐LASSO	0.73	0.076	0.63–0.85	0.24	0.90	0.98	1.06	1.12	1.96
(b.ii) Top 500 empirical covariates using Cox‐LASSO	0.72	0.077	0.62–0.83	0.21	0.89	0.97	1.09	1.13	2.29
(b.iii) Top 100 empirical covariates using RSF	0.72	0.077	0.62–0.83	0.21	0.89	0.97	1.09	1.13	2.29
(b.iv) Top 500 empirical covariates using RSF	0.72	0.077	0.62–0.83	0.21	0.89	0.97	1.09	1.13	2.29
(c) With most relevant data dimensions	0.73	0.076	0.63–0.85	0.26	0.89	0.97	1.07	1.12	2.06
(d) 4‐digit ICD‐9/10 codes	0.73	0.076	0.63–0.85	0.19	0.90	0.98	1.07	1.12	2.08
(e) hdPS model with XGBoost	0.70	0.078	0.60–0.82	0.18	0.87	0.97	1.14	1.15	3.13
(f) Considering SMD ≤ 0.1 as covariate balance	0.72	0.077	0.62–0.84	0.20	0.89	0.97	1.07	1.12	2.04
(B) Nested case control with eight controls per case									
1. Covariate‐adjusted, non‐hdPS analysis^c^	0.74	0.075	0.64–0.86						
2. hdPS with inverse probability weight^b^	0.76	0.075	0.66–0.89	0.20	0.90	0.98	1.05	1.12	1.88
3. Analyses for hdPS parameters^b^									
(a) 200 proxy covariates from each data source	0.75	0.076	0.65–0.87	0.22	0.90	0.98	1.06	1.12	1.99
(b.i) Top 100 empirical covariates using Cox‐LASSO	0.77	0.075	0.66–0.89	0.25	0.90	0.98	1.05	1.12	1.81
(b.ii) Top 500 empirical covariates using Cox‐LASSO	0.77	0.077	0.66–0.89	0.19	0.89	0.97	1.08	1.13	2.24
(b.iii) Top 100 empirical covariates using RSF	0.77	0.075	0.67–0.90	0.22	0.90	0.98	1.06	1.12	1.94
(b.iv) Top 500 empirical covariates using RSF	0.77	0.077	0.67–0.90	0.19	0.89	0.97	1.09	1.14	2.34
(c) With most relevant data dimensions	0.77	0.075	0.66–0.89	0.25	0.90	0.98	1.05	1.12	1.87
(d) 4‐digit ICD‐9/10 codes	0.76	0.076	0.65–0.88	0.21	0.90	0.98	1.06	1.12	1.99
(e) hdPS model with XGBoost	0.76	0.078	0.65–0.88	0.18	0.88	0.98	1.10	1.14	2.50
(f) Considering SMD ≤ 0.1 as covariate balance	0.76	0.075	0.66–0.89	0.20	0.90	0.98	1.05	1.12	1.88
(C) Nested case control with 100 repetitions									
1. Four controls per case									
(a) Covariate‐adjusted, non‐hdPS analysis^c^	0.75	0.074	0.65–0.86						
(b) hdPS with inverse probability weight^b^	0.75	0.071	0.65–0.86	0.21	0.90	0.97	1.07	1.12	2.04
2. Eight controls per case									
(a) Covariate‐adjusted, non‐hdPS analysis^c^	0.75	0.073	0.65–0.87						
(b) hdPS with inverse probability weight^b^	0.74	0.071	0.65–0.85	0.21	0.90	0.98	1.06	1.12	2.00

Abbreviations: CI, confidence interval; hdPS, high‐dimensional propensity score; HR, hazard ratio; RSF, random survival forest; SE, standard error.

^a^ The conditional logistic regression on the matched dataset (matched using incidence density sampling) was fitted, with matched set as the strata variable, adjusting for age and sex.

^b^ Summary of truncated inverse probability weights, with Min indicating minimum, Q1 indicating the first quartile, Q2 indicating the median, Q3 indicating the third quartile, and Max indicating the maximum. Weights were truncated at the 99th percentile.

^c^ The conditional logistic regression on the matched dataset (matched using incidence density sampling) was fitted, with the stabilized inverse probability weight, matched set as the strata variable, adjusting for age. The high‐dimensional propensity score technique was used to estimate the inverse probability weight.

#### 3.4.2 Sensitivity Analyses With Eight Controls Per Case

In the NCC analysis with eight controls per case, 3209 cases were matched to 24 510 controls. While the covariate‐adjusted/non‐hdPS analysis had an HR of 0.74 (95% CI: 0.64–0.86, SE: 0.075), the hdPS resulted in an HR of 0.76 (95% CI: 0.66–0.89). The sensitivity analyses for hdPS parameters also resulted in an HR of 0.75–0.77 with comparable SEs (0.075–0.078) (Table 3B).

#### 3.4.3 Sensitivity Analyses With Repeating NCC Analysis Multiple Times

The distribution of the HRs from the analyses where we repeated the NCC analysis 100 times is presented in Appendix Figure 1. In the 1:4 NCC analysis with 100 repetitions, the non‐hdPS had a pooled HR of 0.75 (95% CI: 0.65–0.86, SE: 0.074). The 1:8 NCC analysis with 100 repetitions also resulted in a pooled HR of 0.75 (95% CI: 0.65–0.85, SE: 0.073). The hdPS analyses resulted in a pooled HR of 0.75 (SE: 0.071) in the 1:4 NCC analysis with 100 repetitions, while it was 0.74 (SE: 0.071) in the 1:8 NCC analysis with 100 repetitions (Table 3C).

#### 3.4.4 Sensitivity Analysis With Time‐Dependent Cox Regression on the Full‐Cohort

In the time‐varying Cox proportional hazards model, adjusting for age and sex, and stratifying by neighborhood income quintile, CCI, and calendar year, we observed a 21% lower risk of mortality associated with any DMD exposure (HR: 0.79, 95% CI: 0.68–0.91, SE: 0.072). The PH assumption was met for age and sex (*p*‐values of 0.537 and 0.477).

## 4 Discussion

### 4.1 Summary of the Findings

We showed how to simultaneously address immortal time bias and residual confounding that are inherent in many observational cohort studies. We used a case study exploring the relationship between DMDs used to treat MS, a time‐dependent exposure, and all‐cause mortality. Using an NCC design with investigator‐specified confounders to address immortal time bias, we showed a 28% lower mortality risk associated with exposure to a DMD used to treat MS. A similar reduction of 28% was observed when we conducted an hdPS analysis within the NCC framework to address residual confounding. We also presented sensitivity analyses across different hdPS parameters and matching strategies for the NCC analyses. We demonstrated analyses with random survival forest instead of Cox‐LASSO to prioritize and select recurrence covariates, alternative propensity score estimation techniques such as XGBoost, four or eight controls per case, and repeating the NCC analysis multiple times with or without hdPS. All these analyses could help practitioners simultaneously address immortal time bias and residual confounding in their analyses. We shared reproducible R codes for researchers to facilitate the adoption of this novel methodology in their research.

### 4.2 NCC to Deal With Immortal Time Bias

Several methods are commonly used to address immortal time bias in observational studies, including the naïve method, prescription time‐distribution matching, and landmark analysis [2, 41, 42, 43]. However, these simpler methods can misclassify the time‐dependent exposure or improperly exclude the immortal time period from the analysis, leading to biased estimates [2, 43]. The NCC and matched‐cohort designs are advanced dynamic matching methods that can address the issue of immortal time bias [28, 44, 45]. Under the proportional hazards assumption, the HR from the full‐cohort time‐varying Cox proportional hazards model and the HR from the NCC or matched‐cohort designs are equivalent [46, 47]. In contrast, the “time‐dependent” exposure becomes a “time‐fixed” exposure in an NCC design, while the exposure remains “time‐dependent” in the full‐cohort time‐varying Cox proportional hazards model or the matched‐cohort design. Thus, implementing hdPS analysis is often more straightforward compared to full‐cohort or matched‐cohort analyses, particularly when handling time‐dependent exposures.

### 4.3 Residual Confounding and the Role of hdPS Within the NCC Framework

To address residual confounding, we employed the hdPS method, which systematically incorporated recurrence covariates from the available data to better control for unmeasured confounding. By applying hdPS within the NCC framework, we treated the time‐varying exposure as a time‐independent variable, simplifying the analysis. Our hdPS‐adjusted findings, which closely aligned with the NCC model results, highlight the utility of this method in improving the validity of treatment effect estimates in observational research. In addition to the NCC design, several approaches can address immortal time bias in observational studies, such as use of the time‐dependent Cox model, sequential Cox, time‐dependent propensity score, target trial emulation, or a matched‐cohort design [28, 43, 44, 45, 48, 49]. Our study does not aim to compare these approaches but rather focuses on demonstrating the feasibility and novelty of applying hdPS within the NCC framework. This combination allows simultaneous handling of immortal time bias and residual confounding bias—an integration not commonly explored in the literature. This approach is particularly valuable in observational studies using linked health administrative data, where residual confounding is often a substantial concern. Further work could assess the generalizability and performance of hdPS across different designs that address immortal time bias.

In the epidemiology literature, the NCC analysis to deal with immortal time bias [50] and hdPS to deal with residual confounding [8, 9] are both familiar approaches. However, the literature demonstrating how to simultaneously deal with immortal time bias and residual confounding is limited. One study we have identified included an application where hdPS with an NCC design was used in exploring the relationship between oral contraceptives and arterial thrombosis [51]. The main differences between the NCC‐hdPS implementation in our study and Larivée et al. [51] lie in the focus and complexity of the analyses. Our study applied Cox‐LASSO, a survival model, to select the Top 200 covariates for the hdPS, while Larivée et al. used a standard Bross‐based hdPS approach with 500 covariates. We conducted more extensive sensitivity analyses, including testing different hdPS parameters and control‐matching strategies (1:4 vs. 1:8), while Larivée et al. primarily tested the robustness of matching on contraceptive use history. Furthermore, our study's time‐varying exposure definition was essential to avoid immortal time bias, a significant issue for long‐term survival analyses, whereas Larivée's focus was on a shorter‐term risk assessment for contraceptive use. Overall, our study addressed more complex longitudinal and survival data with robust methods to handle both bias and confounding.

### 4.4 Contextualize the Findings in the MS Literature

Our results consistently showed that DMD exposure is associated with a significant reduction in all‐cause mortality in MS patients. Our sensitivity analyses across different hdPS parameters and control‐matching strategies demonstrated the stability of our results, with a 27%–30% lower mortality risk associated with MS DMDs. The minimal variation across repetitions and the similarity between hdPS and non‐hdPS results in our study may indicate that measured confounders were sufficient to control for confounding in this particular case. These findings underscore the potential long‐term survival benefits of the DMDs used to treat MS, while also demonstrating the importance of evaluating and applying rigorous statistical methods as appropriate to the observational data context. However, if the empirically identified proxies were poor proxy measures for unmeasured confounders, residual confounding bias might still be present, and hdPS might not effectively mitigate this bias.

Our findings are consistent with several prior studies that have explored the survival benefits of the DMDs used to treat MS [4, 21]. For instance, a previous population‐based study using the same cohort similarly observed a significant reduction in mortality associated with DMD exposure, particularly with the second‐generation DMDs (Appendix C) [4]. However, our results extend previous findings by incorporating more advanced statistical methods to rigorously address biases inherent in observational studies, such as residual confounding through adding proxy confounders. Unlike earlier studies that primarily relied on traditional Cox models, our approach incorporated hdPS analysis within the NCC design, enabling more comprehensive confounder adjustment, including selected proxies. This methodological advancement helps reconcile the varying degrees of survival benefit observed across different studies, highlighting the importance of robust confounding adjustment in this context. Our methodological choices also provide confidence in the validity of the findings and underline the importance of accurately accounting for immortal time bias in studies assessing long‐term outcomes.

### 4.5 Strengths, Limitations, and Future Directions

Several strengths enhance the credibility of our study. First, we leveraged a large, population‐based cohort with a long follow‐up period, allowing us to observe a substantial number of events and enabling robust statistical analyses. Second, our use of advanced methods, including the NCC and hdPS, allowed us to rigorously reduce bias due to both immortal time and residual confounding, issues that may have complicated the interpretation of results in previous studies [4, 7, 20, 52].

Despite these strengths, our study has some limitations. Although the hdPS approach mitigates many of the challenges inherent in using administrative health data by accounting for numerous recurrence covariates, some residual confounding may still remain. In our case study, approximately 17% of people with MS died during the study period. While the HR estimate from the full‐cohort time‐varying Cox proportional hazards model and the NCC analyses is consistent under the proportional hazards assumption, the nonrare event could yield a different HR estimate from the NCC analysis compared to that of a full‐cohort time‐varying Cox proportional hazards model. However, the HR from the NCC analyses was not materially different compared to the full‐cohort time‐varying Cox proportional hazards model. While modeling DMD exposure as a time‐varying covariate can address immortal time bias, it does not fully account for potential differences in prognosis between individuals who initiate treatment early and those who never do. As such, early outcomes may still be differentially attributed, and residual confounding might persist. A recent methodological development utilizing routinely collected health administrative data is target trial emulation [49], which aims to mimic the design and analysis of a hypothetical randomized controlled trial as closely as possible within the constraints of the available observational data [49, 53, 54]. A per‐protocol analysis with a clone‐censor‐weighting approach within this framework could help address the issue of early outcome misclassification among those with a shorter treatment duration group [55]. Future studies could emulate the hypothetical target trial in determining the relationship between DMD exposure and mortality. Dickerman et al. [56] demonstrated how the principles of target trial emulation can be utilized in case–control studies to address immortal time bias. Hernán et al. [57] highlighted the importance of carefully defining time zero, eligibility criteria, and treatment assignment to effectively answer a causal question and address immortal time bias. Although a target trial emulation and implementation of a clone‐censor‐weighting approach can address immortal time bias, future research is needed to examine how residual confounding can be minimized in such frameworks, particularly, when using high‐dimensional covariate data. Additionally, we had access to only four data dimensions to assess recurrence covariates, which may explain the similarity between the non‐hdPS and hdPS results in our case study. Although we used White's robust sandwich estimator to calculate the SE [34], our methods did not account for the uncertainty introduced by the variable selection step; thus, the reported 95% CI may be anticonservative. While IPTW is typically applied in cohort studies, its use in our NCC design is supported by methodological literature under the assumptions of incidence density sampling and a relatively rare outcome. We discuss this rationale in detail in Appendix D, citing relevant works [58, 59, 60, 61, 62], and acknowledge that our estimates reflect marginal effects within the sampled population. Future work could incorporate calibrated sampling weights to formally generalize results to the entire source cohort. Moreover, our study population was limited to British Columbia, Canada, and generalizability of our findings to other regions with different healthcare systems or patient populations remains to be explored. Future research could aim to further refine the hdPS analysis with the NCC framework in different research contexts that include diverse data dimensions for assessing the recurrence covariates. Future simulation studies could explore NCC‐hdPS analysis results by varying key parameters. In the MS context, future studies could focus on exploring the differential effects of individual DMDs, particularly in real‐world settings, where switching between therapies is common [63].

### 4.6 Conclusion

Our study provides a framework for simultaneously dealing with immortal time bias and residual confounding. By employing advanced statistical methods, including hdPS and an NCC design, we addressed key biases inherent in observational studies. A series of sensitivity analyses further reinforced the validity of our findings. Our application of the hdPS technique in the NCC framework showed strong evidence that exposure to a DMD is associated with a significant reduction in all‐cause mortality in MS patients. These results underscore the role of DMDs in improving long‐term survival and emphasize the need for further research to clarify their benefits in MS treatment.

### 4.7 Plain Language Summary

This study looked at how disease‐modifying drugs (DMDs) might help people with multiple sclerosis (MS) live longer. It tackled two common challenges in health research: “immortal time bias,” which can occur when part of a person's follow‐up time is incorrectly counted, and “residual confounding,” which happens when differences between groups are not fully accounted for. The researchers studied over 19 000 MS patients in British Columbia, using a unique approach that combined two advanced statistical methods to address these issues. They found that people taking DMDs had a 28% lower risk of death compared to those who did not take these medications. These results were consistent even after testing the methods in different ways, showing the reliability of their findings. To help other researchers apply these techniques, the team shared step‐by‐step instructions and software tools. This work highlights the important role DMDs can play in extending the lives of people with MS while improving how we study treatments in real‐world settings.

## Author Contributions

Md. Belal Hossain led the formal analysis, software development, and original drafting of the manuscript, and contributed equally to the investigation, methodology, and review. Huah Shin Ng contributed to the validation of the results, provided supporting funding, and contributed equally to the review and editing of the manuscript. Feng Zhu supported funding acquisition, contributed to the validation of the findings, and equally participated in the review and editing of the manuscript. Helen Tremlett shared responsibilities for data curation and resources, provided supporting funding, and contributed equally to the review and editing of the manuscript. Mohammad Ehsan Karim conceptualized the study, supervised the project, led funding acquisition and resources, and contributed equally to the review and editing of the manuscript.

## Ethics Statement

The studies involving human participants were reviewed and approved by the University of British Columbia Clinical Research Ethics Board and British Columbia Ministry of Health (H1800407).

## Consent

Written informed consent to participate in this study was provided by the participants' legal guardian/next of kin.

## Conflicts of Interest

H.S.N. is supported by a Beat Cancer Early Career Research Fellowship from Cancer Council South Australia and has also received funding from the Southern Adelaide Local Health Network Enquiry Grant. Helen Tremlett has, in the last 5 years, received research support from the Canada Research Chair Program, the National Multiple Sclerosis Society, the Canadian Institutes of Health Research, Multiple Sclerosis Canada, the Multiple Sclerosis Scientific Research Foundation, and the EDMUS Foundation (“Fondation EDMUS contre la sclérose en plaques”). In addition, in the last 5 years, has had travel expenses or registration fees prepaid or reimbursed to present at CME conferences or attend meetings (as a member of the International Advisory Committee on Clinical Trials in Multiple Sclerosis) from the Consortium of MS Centres (2023), the Canadian Neurological Sciences Federation (2023), National MS Society (2022, 2023, 2024), ECTRIMS/ACTRIMS (2017–2024), American Academy of Neurology (2019). Speaker honoraria are either declined or donated to an MS charity or to an unrestricted grant for use by H.T.'s research group. M.E.K.'s research is supported by M.E.K.'s Natural Sciences and Engineering Research Council of Canada (NSERC) Discovery Grants and Discovery Accelerator Supplements. M.E.K. was also supported by the Michael Smith Foundation for Health Research Scholar award. Over the past 4 years, Author M.E.K. has received consulting fees from Biogen (unrelated to the current project) and participated in Advisory Boards and/or Satellite Symposia of Biogen Inc. The other authors declare no conflicts of interest.

## Supporting information


**Data S1.** Appendix.

## Data Availability

Access to data provided by the Data Stewards is subject to approval but can be requested for research projects through the Data Stewards or their designated service providers. The following data sets were used in this study: Consolidation file (includes demographics, registry, and census geodata), vital statistics, medical services plan, hospital discharge abstract database, and Pharmanet. You can find further information regarding these data sets by visiting the PopData project webpage at: https://my.popdata.bc.ca/project_listings/18‐120/collection_approval_dates. All inferences, opinions, and conclusions drawn in this publication are those of the author(s), and do not reflect the opinions or policies of the Data Steward(s).
