# Supplementary material for: Simultaneously Dealing With Immortal Time Bias and Residual Confounding: A Case Study of a High‐Dimensional Propensity Score Approach With a Nested Case–Control Framework in Multiple Sclerosis Research
Source: Pharmacoepidemiol Drug Saf. 2025 Jun 24;34(7):e70174. doi: 10.1002/pds.70174 (PMC12186589; doi:10.1002/pds.70174)
Supplement: Supplementary file 1 — Data S1. Appendix. [file PDS-34-e70174-s001.pdf]

Appendix "Simultaneously dealing with immortal time bias and residual confounding: A case study of high-dimensional propensity score approach with a nested case-control framework in multiple sclerosis research"

Table of Contents

Appendix-A: Case study description 2

Multiple sclerosis Cases 2

Appendix-B: Sensitivity analyses 2

XGBoost 2

Modified multiple outputation technique 2

Time-dependent Cox regression on the full-cohort 2

Appendix-C: Exploring the relationship between DMDs and mortality 4

Multiple Sclerosis and Disease-Modifying Drugs 4

Long-Term Impact of DMDs on Survival 4

Appendix-D: Use of inverse probability of treatment weighting in a nested case-control design . 5

Appendix Tables 7

Appendix Table 1: Characteristics of the study participants where each case was matched up to four controls using nested-case control design. Here, the case indicates all-cause mortality events. 7

Appendix Figures 8

Appendix Figure 1: Distribution of the hazard ratios from 100 repetitions of the nested case-control analyses in exploring the relationship between exposure to any disease-modifying drug (DMD) for multiple sclerosis and all-cause mortality in British Columbia, Canada, 1996-2017. Each case (all-cause mortality event) was matched up to (a) four and (b) eight controls. 8

Appendix Reference 9

Appendix-A: Case study description

Multiple sclerosis Cases

We identified individuals with multiple sclerosis (MS) using information from physician visits, hospitalizations, and prescriptions filled. A validated algorithm was used that required ≥ 3 MS-specific physician visits and/or hospitalizations with ICD-9/10 codes 340/G35, or one prescription filled for an MS disease-modifying drug (DMD) ever [1,2].

Appendix-B: Sensitivity analyses

XGBoost

XGBoost is an ensemble learning algorithm based on gradient boosting, which builds a series of decision trees. Each new tree is trained to correct the errors made by the previous trees [3]. This iterative process allows XGBoost to model complex, non-linear relationships and interactions among covariates. Because of these strengths, XGBoost may offer advantages in hdPS applications by more effectively capturing relationships among both investigator-specified and high-dimensional empirical covariates.

Modified multiple outputation technique

To account for the precision improved in the hazard ratio (HR) estimate due to repeated samplings, the modified multiple outputation technique was used [4–6]. The following equation was used to calculate the model-based standard error (SE) for the analyses with repeated samplings:

$$\mathrm{SE} = \sqrt{\mathrm{median}\left(\left|V_w - \left(\hat{\beta} - \tilde{\beta}\right)^2\right|\right)},$$

where V_w is the within variance in the log-HRs, $\hat{\beta}$ is the estimated log-HRs, and $\tilde{\beta}$ is the median of $\hat{\beta}$'s.

Time-dependent Cox regression on the full-cohort

We conducted a sensitivity analysis with full-cohort time-varying Cox proportional hazards model that helps mitigate immortal time bias but not residual confounding. For that analysis, we used a time-varying exposure definition to avoid issues with immortal time bias [7]. For those who were unexposed, their time contribution was from the index date to the event date or censoring (due to emigration or study end). For those who were exposed to any DMD, the unexposed time was the time contributed from the index date up to the DMD exposure, and the exposed time began at the date of DMD exposure and continued until the event date or censoring was reached. As done in a previous study [1], we first fitted stratified TD-Cox regression on the full-cohort with calendar year as a strata variable, adjusting for age, sex, neighborhood income quintile, and the Charlson comorbidity index (CCI). The proportional hazards assumption was evaluated using Schoenfeld residuals, and violations for neighborhood income quintile and the CCI were identified, likely due to changing effects of these variables over time. Hence, we considered neighborhood income quintile, CCI, and calendar year as strata variables in the TD-Cox regression, adjusting for age and

sex. The proportional hazards assumption was met for the final model, which resulted in an HR of 0.79 (95% CI: 0.68-0.91).

Appendix-C: Exploring the relationship between DMDs and mortality

Multiple Sclerosis and Disease-Modifying Drugs

MS is a chronic, immune-mediated disease that targets the central nervous system (CNS), leading to a range of disabling physical and cognitive impairments. Over the past few decades, DMDs have become a cornerstone in MS management, primarily by reducing the frequency of relapses and slowing the progression of disability. However, despite their widespread adoption, the long-term effects of DMDs, particularly in relation to overall survival, remain an area of active research. Gaining a clear understanding of these effects is important for optimizing treatment strategies and improving long-term outcomes for MS patients.

Long-Term Impact of DMDs on Survival

Randomized controlled trials (RCTs) have been essential in demonstrating the short-term efficacy of the DMDs used to treat MS. However, these trials typically span only 2–3 years, a period insufficient to assess long-term outcomes such as overall survival [8,9]. Furthermore, these RCTs often excluded older individuals and those with comorbidities, limiting the generalizability of findings to real-world MS populations [9]. Observational studies have attempted to fill this gap, with some showing that DMD exposure is associated with reduced mortality. For example, one large population-based study from our group found a 26% reduction in mortality among those exposed to any DMD, with a 33% reduction observed for second-generation DMDs [8]. However, the survival benefits varied over time, with some DMDs, such as beta-interferon and glatiramer acetate, showing diminishing effects in the long term [8]. This emphasizes the need for more robust, long-term evidence to confirm these associations.

Appendix-D: Use of inverse probability of treatment weighting in a nested case-control design

Inverse probability of treatment weighting (IPTW) is a widely used method for estimating marginal causal effects in cohort studies. However, its application in a nested case-control (NCC) design requires careful consideration, because NCC samples are drawn with outcome-dependent sampling—cases are typically oversampled, and controls are selected from the risk set. This may result in biased estimates if the sampling structure is not addressed appropriately.

In our study, we used an NCC design with incidence density sampling and included nearly all deaths from the underlying cohort (3,209 of 3,210). This is important because, under incidence density sampling and the proportional hazards assumption, hazard ratios estimated using stratified or weighted models can remain valid for the full cohort. Our use of IPTW, based on a high-dimensional propensity score (hdPS) – which incorporated investigator-specified covariates and a large number of empirical covariates representing proxy information, aims to estimate marginal effects by reweighting individuals according to their estimated treatment probabilities. In doing so, we depart from the traditional conditional logistic regression approach and instead adopt a marginal structural modeling framework.

The appropriateness of IPTW in NCC designs has been addressed in several key methodological papers. Månsson et al. [10] caution that naïve use of propensity scores in case-control settings may lead to residual confounding or spurious associations, especially in small samples or when the outcome is common. However, in large samples and with rare outcomes, these issues tend to diminish. VanderWeele and Vansteelandt [11] show that, under a rare-outcome assumption, causal effects can be validly estimated from a case-control study using IPTW, provided the exposure model is properly specified and estimated among controls. Given the relatively low outcome incidence (~7.8%) and the use of incidence density sampling, the exposure model estimated from the sampled controls is expected to approximate that of the full cohort. Under these conditions, IPTW can yield approximately unbiased marginal effect estimates without requiring explicit sampling weights [11]. Van der Laan [12] further demonstrates that when the outcome prevalence is known (even when the outcome is not extremely rare), estimates from IPTW (or Targeted Maximum Likelihood Estimator) can be calibrated to reflect population-level effects.

Borgan et al. [13] provide additional justification by showing that weighting methods can be used in NCC studies to "break the matching" and recover marginal effects, as long as the design is properly accounted for. In our case, the rare outcome, the use of incidence density sampling, and the near-complete inclusion of cases reduce the need for additional design-based sampling weights. Our IPTW approach, therefore, provides approximately unbiased marginal effect estimation for the underlying cohort.

To assess robustness, we conducted a sensitivity analysis where the propensity score model included the matching criteria (calendar year, age group, and region) as covariates. This adjustment partially accounts for the sampling design. The resulting hazard ratios (0.72–0.73) and standard

errors (0.076–0.078) were comparable to those from the main analysis, suggesting that the IPTW results are not sensitive to this modeling choice.

Nonetheless, we acknowledge that our IPTW implementation does not incorporate explicit sampling weights based on the probability of being selected as a control. Thus, while our estimates reflect marginal effects within the sampled population, we cannot claim full generalizability to the entire source cohort. Future work could incorporate calibrated weights or known outcome prevalence to further refine marginal effect estimates, as recommended in the methods of Cole et al. [14] and van der Laan [12].

Appendix Tables

Appendix Table 1: Characteristics of the study participants where each case was matched up to four controls using nested-case control design. Here, the case indicates all-cause mortality events.

Characteristics	Total (N = 15,463)	Case (N = 3,209)	Matched control (N = 12,254)
Any DMD	2,413 (15.6)	228 (7.1)	2,185 (17.8)
Age in years, mean (SD)	47.59 (13.60)	57.28 (14.33)	45.05 (12.19)
Sex			
Female	11,107 (71.8)	1,969 (61.4)	9,138 (74.6)
Male	4,356 (28.2)	1,240 (38.6)	3,116 (25.4)
Neighborhood income			
Lowest 20%	3,507 (22.7)	729 (22.7)	2,778 (22.7)
Lower 20%	3,154 (20.4)	655 (20.4)	2,499 (20.4)
Middle 20%	3,084 (19.9)	639 (19.9)	2,445 (20.0)
Higher 20%	3,065 (19.8)	634 (19.8)	2,431 (19.8)
Highest 20%	2,653 (17.2)	552 (17.2)	2,101 (17.1)
Charlson comorbidity index			
0	10,713 (69.3)	2,216 (69.1)	8,497 (69.3)
1	2,759 (17.8)	576 (17.9)	2,183 (17.8)
2	1,158 (7.5)	244 (7.6)	914 (7.5)
≥3	833 (5.4)	173 (5.4)	660 (5.4)
Calendar index			
1996-1999	11,517 (74.5)	2,377 (74.1)	9,140 (74.6)
2000-2005	2,278 (14.7)	477 (14.9)	1,801 (14.7)
2006-2011	1,286 (8.3)	271 (8.4)	1,015 (8.3)
2012-2017	382 (2.5)	84 (2.6)	298 (2.4)

DMD: disease-modifying drug; SD: standarad deviation.

Appendix Figures

Appendix Figure 1: Distribution of the hazard ratios from 100 repetitions of the nested case-control analyses in exploring the relationship between exposure to any disease-modifying drug (DMD) for multiple sclerosis and all-cause mortality in British Columbia, Canada, 1996-2017. Each case (all-cause mortality event) was matched up to (a) four and (b) eight controls.

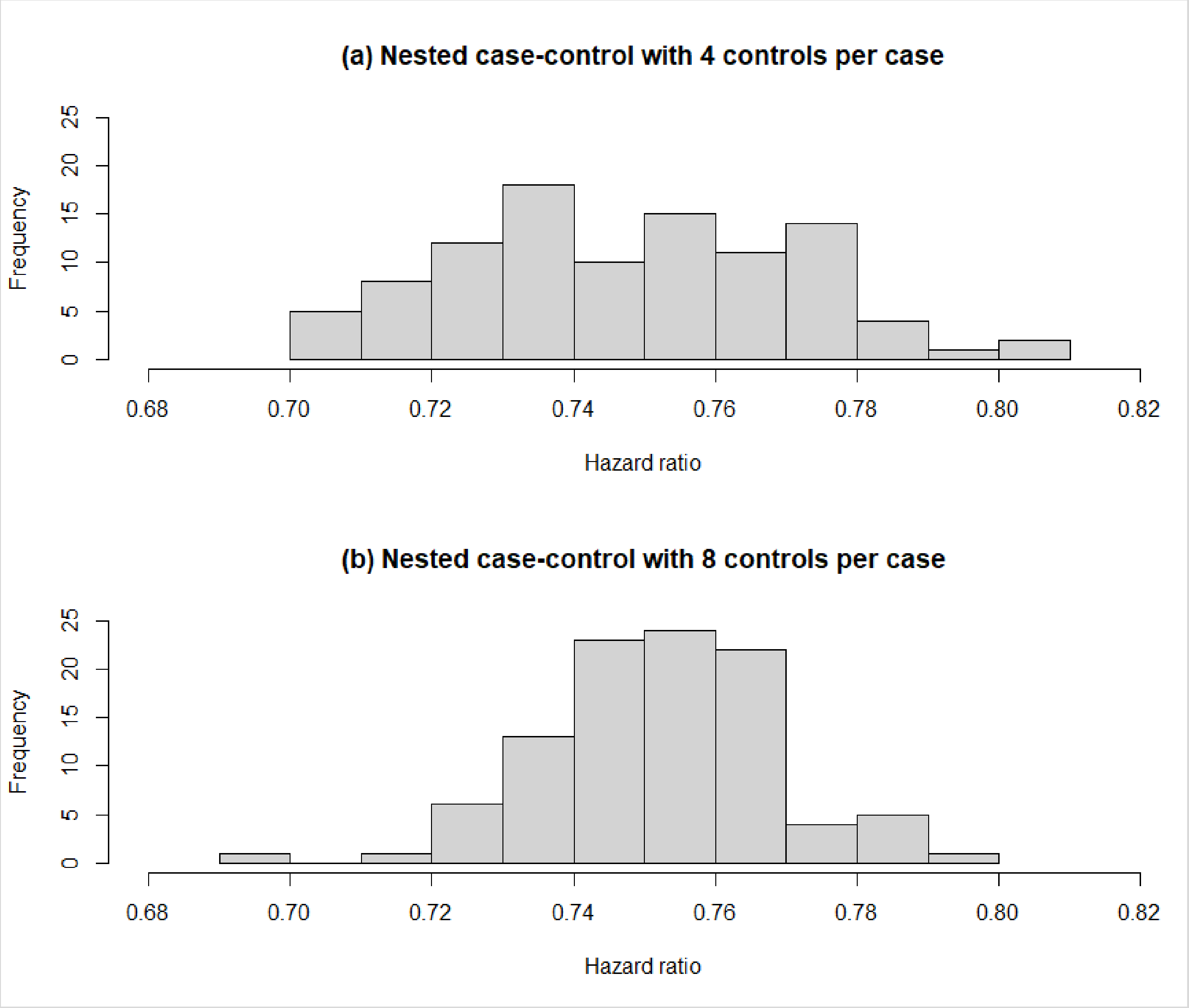

Appendix Reference

[1] Ng HS, Zhu F, Kingwell E, Yao S, Ekuma O, Evans C, et al. Disease-Modifying Drugs for Multiple Sclerosis and Association With Survival. Neurol Neuroinflammation 2022;9:e200005.

[2] Marrie RA, Yu N, Blanchard J, Leung S, Elliott L. The rising prevalence and changing age distribution of multiple sclerosis in Manitoba. Neurology 2010;74:465–71.

[3] Karim ME, Petkau J, Gustafson P, Tremlett H, Group TBS. On the application of statistical learning approaches to construct inverse probability weights in marginal structural cox models: hedging against weight-model misspecification. Commun Stat Comput 2017;46:7668–97.

[4] Hossain MB, Wong H, Sadatsafavi M, Johnston JC, Cook VJ, Karim ME. Benefits of Repeated Matched-Cohort and Nested Case-Control Analyses with Time-dependent Exposure in Observational Studies. Stat Biosci 2024.

[5] Follmann D, Proschan M, Leifer E. Multiple outputation: inference for complex clustered data by averaging analyses from independent data. Biometrics 2003;59:420–9.

[6] Zivich PN, Breskin A. Machine learning for causal inference: on the use of cross-fit estimators. Epidemiology 2021;32:393–401.

[7] Zhou Z, Rahme E, Abrahamowicz M, Pilote L. Survival bias associated with time-to-treatment initiation in drug effectiveness evaluation: a comparison of methods. Am J Epidemiol 2005;162:1016–23.

[8] Ng HS, Zhu F, Kingwell E, Zhao Y, Yao S, Ekuma O, et al. Disease-modifying drugs for multiple sclerosis and subsequent health service use. Mult Scler J 2022;28:583–96.

[9] Ng HS, Zhu F, Zhao Y, Yao S, Lu X, Ekuma O, et al. Adverse Events Associated With Disease-Modifying Drugs for Multiple Sclerosis: A Multiregional Population-Based Study. Neurology 2024;102:e208006.

[10] Månsson R, Joffe MM, Sun W, Hennessy S. On the estimation and use of propensity scores in case-control and case-cohort studies. Am J Epidemiol 2007;166:332–9.

[11] VanderWeele TJ, Vansteelandt S. A weighting approach to causal effects and additive interaction in case-control studies: marginal structural linear odds models. Am J Epidemiol 2011;174:1197–203.

[12] van der Laan MJ. Estimation based on case-control designs with known prevalence probability. Int J Biostat 2008;4.

[13] Borgan Ø, Breslow N, Chatterjee N, Gail MH, Scott A, Wild CJ. Handbook of statistical methods for case-control studies. CRC Press; 2018.

[14] Cole SR, Hudgens MG, Tien PC, Anastos K, Kingsley L, Chmiel JS, et al. Marginal structural models for case-cohort study designs to estimate the association of antiretroviral therapy initiation with incident AIDS or death. Am J Epidemiol 2012;175:381–90.
